# Association Between Increased Dietary Sodium Intake and Higher Water Intake from Fluid and Food in Children

**DOI:** 10.3390/nu17071099

**Published:** 2025-03-21

**Authors:** Yi Zhang, Yongye Song, Shuai Cheng, Yunting Xia, Hongxing Li, Jiangping Huang, Luxi Xu, Na Zhang

**Affiliations:** 1 Department of Nutrition and Food Hygiene, School of Public Health, Peking University, 38 Xue Yuan Road, Haidian District, Beijing 100191, China; 2010108611@stu.pku.edu.cn (Y.Z.); songyongye@bjmu.edu.cn (Y.S.); 2 National Center for Rural Water Supply Technical Guidance, Chinese Center for Disease Control and Prevention, Beijing 102200, China; s18235669176@163.com (S.C.); xiayt@ncrwstg.chinacdc.cn (Y.X.); lihx@ncrwstg.chinacdc.cn (H.L.); 3 Institute of Environmental Hygiene and Endemic Disease Control, Guangxi Zhuang Autonomous Region Center for Disease Control and Prevention, Nanning 530028, China; hjpxiaosa@163.com (J.H.); xuluxi468@126.com (L.X.); 4 Laboratory of Toxicological Research and Risk Assessment for Food Safety, Peking University, 38 Xue Yuan Road, Haidian District, Beijing 100191, China

**Keywords:** sodium intake, hydration status, water intake from fluid, water intake from food, children

## Abstract

**Background and Objectives**: Maintaining an appropriate hydration status is crucial for promoting health. Children, who are in the process of growth and development, are at a higher risk of insufficient water intake and dehydration. This study aimed to compare water intake among children with different levels of dietary sodium intake, and explore the relationship between hydration status, and dietary sodium intake and water intake. **Methods:** In this cross-sectional survey, 155 students in grades 4–6 from a primary school in Binyang County, Nanning, Guangxi, were recruited. Water intake from fluid was assessed using a validated 7-Day 24 h Fluid Intake Survey Questionnaire (days 1–7). Food intake was recorded and weighed using the duplicate diet method on days 5, 6, and 7. The water content in food was determined using the direct drying method, and dietary sodium intake was measured using inductively coupled plasma-optical emission spectrometry (ICP-OES). Urine osmolality was measured at two time points (morning and before afternoon classes) on days 5, 6, and 7 to assess hydration status. **Results:** A total of 155 participants (87 boys and 68 girls) completed the study, with a completion rate of 100%. The average dietary sodium intake, total water intake (TWI), water intake from fluid, and water intake from food were 1647 mg, 2039 mL, 956 mL, and 1175 mL, respectively. Among the participants, 19.4% exceeded the recommended sodium intake (2000 mg/day), 41.9% did not meet the adequate daily water intake from fluid, and 63.2% did not meet the adequate daily total water intake. When participants were divided into quartiles based on dietary sodium intake, significant differences were observed in water intake from fluid (*p* = 0.031) and food (*p* < 0.001). The water intake from fluid among participants in the HS1 (982 mL) and HS2 groups (997 mL) was higher than that among participants in LS2 (759 mL). Water intake from food increased progressively with increasing sodium intake (851 mL, 1075 mL, 1224 mL, and 1550 mL). Urine osmolality was associated with meeting the daily adequate water intake from fluid (*p* = 0.006), but not with exceeding the sodium intake standard (*p* = 0.787). There was no interaction between meeting the daily adequate water intake from fluid and exceeding the sodium intake standard (*p* = 0.413). **Conclusions:** Insufficient water intake was common among children. Children with a higher dietary sodium intake had a higher water intake from fluid and food. Urine osmolality was closely related to daily water intake from fluid, but not to sodium intake.

## 1. Introduction

Water is essential for sustaining life, and is an important component of the human body, performing a variety of physiological functions. The water content in the human body is in a dynamic equilibrium, meaning that water intake and output are balanced, otherwise normal physiological functions and overall health may be compromised. Insufficient water intake or excessive water loss can lead to dehydration, which affects cognitive ability and physical performance, and increases the risk of urinary system diseases [1,2,3]. Conversely, excessive water intake that exceeds the kidneys’ excretory capacity (0.7–1.0 L/h) may cause acute water intoxication. Maintaining an appropriate hydration status is of great significance for maintaining and promoting health [4].

The maintenance of an appropriate hydration status is primarily regulated by a complex neuro-humoral-endocrine network. Sodium (Na⁺) plays a pivotal role in water and electrolyte metabolism. As the principal cation in the extracellular fluid, an increase in sodium concentration leads to elevated osmotic pressure, which, in turn, activates neural signaling pathways. When blood sodium concentration increases or blood volume is reduced, renin secretion increases, activating the renin–angiotensin–aldosterone system (RAAS). This activation promotes the reabsorption of sodium ions in the renal tubules, which, in turn, drives the reabsorption of water, thereby maintaining the body’s water and electrolyte balance [5].

According to the Dietary Guidelines for Chinese School-Aged Children (2022), school-aged children are defined as minors aged 6 years to under 18 years [6]. This period is characterized by rapid growth and development, making it crucial to establish healthy water intake behaviors to support their healthy development. However, the importance of water is often overlooked, with children’s knowledge of healthy drinking practices being incomplete and sometimes misguided. Surveys have revealed significant gaps in children’s hydration knowledge and practices. A study of 1049 children in London found that their knowledge of water intake and health was low, with an average score of only 40% on a hydration knowledge scale [7]. Similarly, a survey of 586 adolescent athletes in Singapore showed that their average correct rate of hydration knowledge was 44.7%, well below the desired 80% [8]. These knowledge deficits directly impact children’s water intake behaviors, leading to widespread insufficient water intake. Compared to adults, children have a relatively larger body surface area [9], higher body water content, and higher levels of daily physical activity. Their thirst-regulating nervous system is also less mature [10]. These factors collectively increase their susceptibility to dehydration. A national survey in China in 2011 found that 65.4% of 5868 primary and secondary school students did not meet the recommended daily water intake of 1200 mL set by the Chinese Residents’ Dietary Guidelines 2016 [11]. Similar findings have been reported globally. In Spain, a 2012 survey of 238 children aged 3 to 17 years showed that 87% had water intakes below 80% of the recommended water intake set by European Food Safety Authority’s (EFSA) [12]. A cross-national survey across 13 countries revealed that 61% of children aged 4 to 9 years and 75% of adolescents aged 10 to 17 years did not meet EFSA’s recommended water intake [13]. In Belgium, a 2012 survey of 1045 schoolchildren found that 90.5% did not achieve adequate water intake [14]. In Lebanon, a 2014 survey of 1209 children showed that 74% of children aged 4 to 8 years and 92% of children aged 9 to 13 years did not meet the recommended water intake [15]. Among adolescent football players, a study using urine-specific gravity and percentage of body weight loss to assess hydration status found that 79 athletes experienced dehydration before and after training, with an average weight loss of 0.7 ± 0.7% [16]. Given the prevalence of inadequate water intake and the potential health risks associated with dehydration, it is essential to focus on promoting healthy water intake behaviors among children to ensure they maintain an appropriate hydration status.

The relationship between dietary sodium intake and water intake in children remains a topic of ongoing debate. Generally, an increased sodium intake raises the osmotic pressure of the extracellular fluid, stimulating thirst to maintain fluid balance. For instance, a study of 6400 children aged 2–18 years in U.S. found a positive correlation between dietary sodium intake and water intake [17]. Similarly, a cross-sectional analysis of 1688 British children aged 4–18 years showed that salt was a major determinant of children’s water intake [18]. However, clinical studies have suggested that variations in salt intake ranging from 0.6 g/d to 24 g/d may not alter fluid intake or urine volume in humans [19]. In addition, an experimental study involving 10 healthy males found that increased salt intake promoted water conservation in the body and reduced fluid intake [20]. Whether differences in dietary sodium intake affect children’s total water intake (TWI) or water intake from food remains to be further investigated. Moreover, studies reporting the relationship between dietary sodium intake, water intake, and hydration status are limited and yield inconsistent results. A randomized crossover study exploring the relationship between hydration status, thirst, and salt preference in adults revealed that the hydration status could influence salt cravings and the consumption of foods with varying water contents [21]. In contrast, an analysis based on the DONALD cohort study showed that salt intake did not affect the hydration status in children [22].

The present study investigated children’s dietary sodium intake and water intake, aiming to compare differences in water intake among children with varying levels of dietary sodium intake. Additionally, the study sought to explore the relationship between children’s hydration status, and their dietary sodium intake and water intake, providing evidence for health education related to sodium and water intake in children.

## 2. Study Subjects and Methods

### 2.1. Study Subjects

Sample Size Calculation: In this cross-sectional survey, fourth- to sixth-grade boarding elementary school students were recruited from Binyang, a rural county in Guangxi, China. The sample size was calculated using the following formula: N = (Z_1-−α/2_σ/δ)^2^, where α = 0.05, Z_1−α/2_ = 1.96, the estimated standard deviation (σ) was 434 mL [23] and the allowable error (δ) was 76 mL [24] based on previous studies. Considering a 15% dropout rate and sampling error, the final calculated sample size was 147 participants.

Participants: A total of 155 fourth-to-sixth-grade boarding elementary school students were recruited for this study. The study was approved by the Ethics Review Committee of National Center for Rural Water Supply Technical Guidance, Chinese Center for Disease Control and Prevention (approval no. 2023002), and informed consent was obtained from all participants. Inclusion Criteria: Healthy fourth-to-sixth-grade boarding elementary school students. Exclusion Criteria: Students with oral, endocrine, renal, cardiac, gastrointestinal, metabolic diseases, or other chronic conditions; cognitive impairments; alcohol consumption (>20 g/day); or coffee intake (>250 g/day) [25].

### 2.2. Study Methods

#### 2.2.1. Physical Examination

Anthropometric measurements were taken twice at the same time point for each subject, and the average value was used as the final result. The methods for each measurement were as follows:

Height was measured by trained project staff. Participants stood barefoot, without hats or thick outerwear, with heels, sacrum, and interscapular region in contact with the stadiometer column. The head was positioned upright, with the eyes looking straight ahead and the upper edge of the ear screen aligned with the lower edge of the orbit. Height was recorded in centimeters (cm) to the nearest 0.1 cm, and the average of the two measurements was used.

Weight was measured by trained project staff using a mechanical scale. The scale was calibrated daily before use. Participants stood barefoot and in light clothing, with feet flat on the scale platform without touching any other objects. Weight was recorded in kilograms (kg) to the nearest 0.1 kg, and the average of two measurements was used.

#### 2.2.2. Temperature and Humidity Measurement

The temperature and humidity of the indoor and outdoor environments (cafeteria, dormitory, and classroom) in Nanning, China, were recorded at 10:00, 14:00, and 20:00 daily for seven consecutive days. Temperature was measured to the nearest 0.1 °C, and humidity was measured to the nearest 1%. The temperatures of indoors and outdoors during the 7 days were 19.9 °C and 19.7 °C, respectively. The humidity was 70.3% and 69.0%, respectively (see Supplementary Table S1).

#### 2.2.3. Water Intake from Fluid Survey

Water intake from fluid was assessed using a self-designed 7-Day 24-h Fluid Intake Survey Questionnaire, which has been validated for reliability and validity in previous studies [24,26,27]. Participants were provided with standardized measuring cups (with graduations accurate to 5 mL) and instructed to record the type, volume (mL), time, and location of each water intake behavior over seven days under the guidance of investigators.

#### 2.2.4. Dietary Sodium Intake and Water Intake from Food Survey

Dietary sodium intake and water intake from food were assessed over three consecutive days using a combination of weighing and duplicate diet methods. Participants were required to cooperate with researchers to weigh and record food items using an electronic balance before consumption. During meals, participants were instructed not to mix different types of food or discard inedible parts (e.g., bones, peels). Post-meal weighing procedures were identical to pre-meal procedures. Researchers were responsible for weighing, recording, and calculating actual intake based on pre- and post-meal data.

Food samples were sealed and refrigerated before being sent for analysis. Water content in food was determined using the direct drying method according to the GB 5009.3-2016 National Food Safety Standard for the Determination of Moisture in Food [28], and sodium content was measured using inductively coupled plasma-optical emission spectrometry (ICP-OES) according to the GB 5009.268-2016 National Food Safety Standard for the Determination of Multiple Elements in Food [29].

For fruits and snacks, trained investigators used an electronic balance to weigh the items before and after consumption over three consecutive days. Water and sodium intake from fruits and snacks were calculated based on actual consumption and the water and sodium content ratios provided in the Chinese Food Composition Table (6th Edition) [30].

Total water intake (mL) = water intake from fluid (mL) + water intake from food (mL)

#### 2.2.5. Urine Osmolality Survey

Urine samples were collected from students at two time points (morning and before afternoon classes) over three consecutive days. Participants were instructed not to drink large amounts of water in the half-hour preceding urine collection. Clean, dry, disposable urine cups were used to collect midstream urine samples (approximately 10–12 mL), which were then transferred to disposable urine tubes. Urine samples were collected to avoid contamination with feces, menstrual blood, or other impurities, and female participants were instructed to avoid sampling during menstruation. Two full-process blank samples were collected daily.

Urine samples were refrigerated at 2–8 °C if not tested immediately, kept away from heat sources, and protected from light. Samples were tested within 8 h of collection. Urine osmolality was measured using the freezing point depression method according to the 2019 Edition of the Chinese Pharmacopoeia Standard Operating Procedure for Osmolality Determination [31]. The hydration status was assessed based on urine osmolality: ≤500 mOsm/kg indicated adequate hydration; 500–800 mOsm/kg indicated borderline hydration; and >800 mOsm/kg indicated dehydration [23,32].

### 2.3. Quality Control

Prior to the field survey, a standardized protocol manual was developed, and uniform survey forms and methods were used. All surveyors were trained to ensure consistency. To ensure the accuracy and compliance of the 7-Day 24 h Fluid Intake Survey Questionnaire, surveyors reviewed the questionnaires daily, communicated with participants to address any issues, and reported to the researchers. During the 3-day 24 h dietary survey, all food items were weighed and recorded before and after consumption. For urine collection, surveyors verified each participant’s urine records for correct labeling and timing. Urine samples were checked for correct labeling during testing. Surveyors verified all urine indicators daily to ensure data completeness and accuracy.

### 2.4. Statistical Analysis

Statistical analysis was performed using SPSS version 26.0. For normally distributed data, the mean ± standard deviation was used to describe central tendency and dispersion. A one-way ANOVA or t-test was used to compare indicators among different groups, with Student–Newman–Keuls (SNK) post hoc tests for pairwise comparisons (*p* < 0.05). For skewed data, the median and interquartile range were used to describe central tendency and dispersion. Differences among participants with different characteristics were compared using χ^2^ tests, Wilcoxon rank-sum tests, or Kruskal–Wallis tests. Statistical significance was set at *p* < 0.05.

## 3. Results

### 3.1. Basic Characteristics

A total of 155 participants were recruited, including 87 boys and 68 girls, with a completion rate of 100%. The median age of the participants was 11 years, with an average height of 144 cm and an average weight of 36.5 kg. Among the participants, 20 children were underweight and 32 children were overweight (Table 1). The BMI limit values of children of different genders and ages are presented in Supplementary Table S2.

### 3.2. Sodium and Water Intake

**Figure 1:**
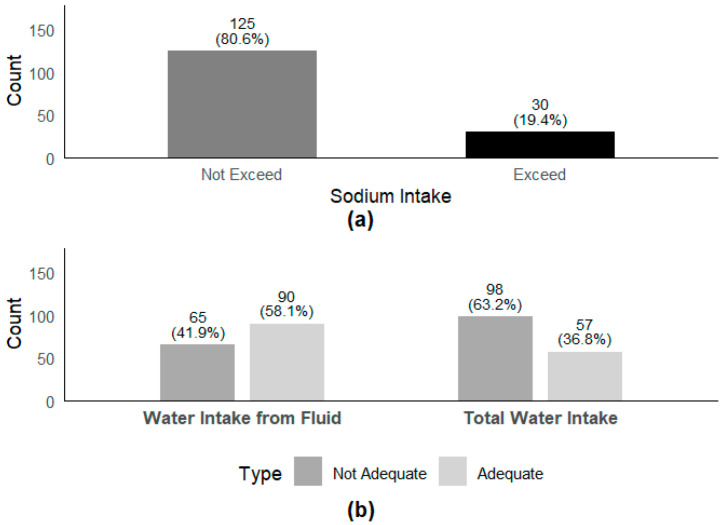
Sodium intake exceeded the recommended amount, water intake from fluid and total water intake reached the recommended amount. (**a**) Dietary sodium intake exceeding the recommended amount by the World Health Organization (2 g/day); (**b**) water intake from fluid and total water intake meeting the adequate daily water intake from fluid (1000 mL for children aged 7–11 years; 1300 mL for boys and 1100 mL for girls aged 12–14 years) [6], and the adequate daily total water intake (1800 mL for children aged 7–11 years; 2300 mL for boys and 2000 mL for girls aged 12–14 years) [33].

The average daily intake of dietary sodium, total water intake, water intake from fluid, and water intake from food among the participants were 1647 mg, 2039 mL, 956 mL, and 1175 mL, respectively (Table 2). According to the classification of water intake from fluid types, the median intake of plain water is 805 mL, milk is 18 mL, while tea and sugary drinks are all 0 mL. Non-compliance with recommended intake levels was widespread. Specifically, 19.4% of the participants exceeded the recommended dietary sodium intake, 41.9% did not meet the adequate daily water intake from fluid, and 63.2% did not meet the adequate daily total water intake (Figure 1).

**Figure 2:**
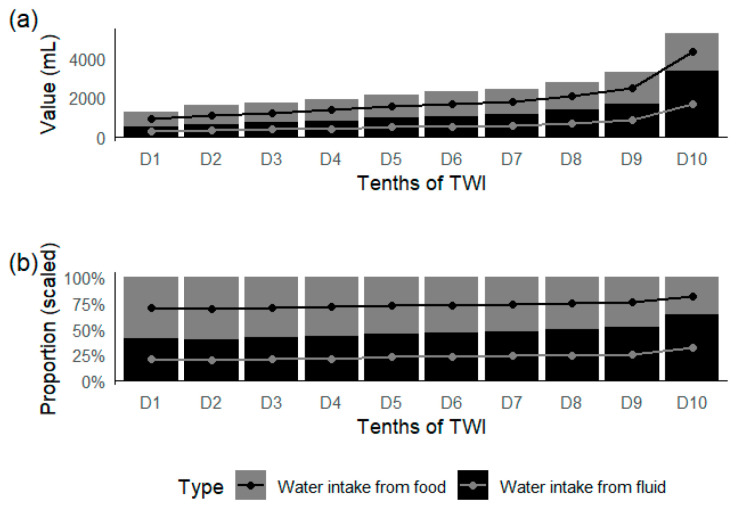
Volume (**a**) and contribution (**b**) to total water intake of water intake from food and fluid.

**Figure 3:**
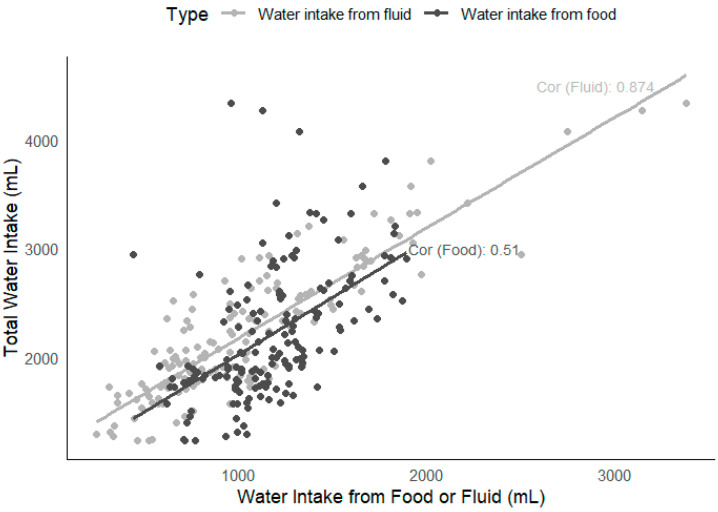
The relationship between TWI and water intake from food or fluid.

Figure 2 shows the proportion of water intake from food and fluid in TWI. With an increase in TWI, the water intake from fluid increased from 522 mL to 3382 mL, and the proportion of water intake from fluid increased from 40.7% to 64.1%. The correlation between TWI and water intake from fluid (R^2^ = 0.874) was higher than that between TWI and water intake from food (R^2^ = 0.51) (Figure 3). The results showed that 87.4% of the variance in TWI was explained by the difference in water intake from fluid.

As seen in Table 2, there were statistically significant differences in dietary sodium intake and water intake from food between different genders (*p* < 0.001). Compared with males, females had an increase of 198 mg in dietary sodium intake and an increase of 293 mL in water intake from food. There were also statistically significant differences in the proportion of water intake from fluid and the proportion of water intake from food between different genders (*p* = 0.001), with females having a higher proportion of water intake from fluid (49%) than males (42%). However, there were no significant differences in dietary sodium intake and water intake among children classified by different BMI categories (*p* > 0.05).

### 3.3. Relationship Between Dietary Sodium Intake and Water Intake

Participants were split into four groups: low sodium intake 1 (LS1), low sodium intake 2 (LS2), high sodium intake 1 (HS1), and high sodium intake 2 (HS2) groups, according to the quartiles of sodium intake of the participants (Q1, <1319 mg; Q2, 1320–1585 mg; Q3, 1586–1924 mg; Q4, >1924 mg).

As seen in Table 3, there were differences in dietary sodium intake between different genders (*p* = 0.001). The proportion of boys in the high-sodium intake groups (HS1, 70.00%; HS2, 71.05%) was higher than that in the low-sodium intake group (LS1, 31.58%), while the proportion of girls was lower. There were differences in water intake from fluid (*p* = 0.031) and food (*p* < 0.001) among different levels of dietary sodium intake. The water intake from fluid among participants in HS1 (982 mL) and HS2 groups (997 mL) was higher than that among participants in LS2 (759 mL). The water intake from food increased progressively with increasing dietary sodium intake (851 mL, 1075 mL, 1224 mL, 1550 mL). There were differences in the TWI and the proportion of TWI meeting the adequate intake among different levels of dietary sodium intake (*p* < 0.001). Compared with the LS1 and LS2 groups, the HS1 and HS2 groups had a higher TWI and a higher proportion of TWI meeting the adequate intake. There were differences in the proportion of water intake from fluid to TWI among different levels of dietary sodium intake (*p* < 0.001). The proportion of water intake from fluid in the LS2 group (42%) and HS2 group (39%) was lower than that in the LS1 group (52%).

### 3.4. Relationship Between Hydration Status and Dietary Sodium Intake and Water Intake

The daily average urine osmolality of participants met the normality test, with a mean of 391 ± 114 (mOsm/kg). An analysis of variance was conducted with urine osmolality as the dependent variable and whether the fluid intake standard was met and whether the dietary sodium intake exceeded the recommended amount as independent variables. The results showed no interaction between meeting the fluid intake standard and exceeding the recommended dietary sodium intake (*p* = 0.413). Urine osmolality was associated with whether the fluid intake standard was met (*p* = 0.006), but not with whether the dietary sodium intake exceeded the recommended amount (*p* = 0.787).

## 4. Discussion

The results indicate that excessive dietary sodium intake, insufficient water intake from fluid, and inadequate total water intake were prevalent among children, consistent with findings from multiple studies, both domestically and internationally. A study on sodium excretion in Chinese children revealed that the average dietary sodium intake among 8-to-9-year-olds was 2181 mg/day, with 77% exceeding the recommended intake for their age group in the Chinese Residents’ Dietary Guidelines (≤4 g/day) and even surpassing the WHO’s recommendation for healthy adults (≤5 g/day) [34]. Another study analyzed the sodium intake trends among American children and adolescents from 2003 to 2016. It found that although there was a declining trend in sodium intake, a significant number of children still consumed excessive amounts of sodium [35]. Additionally, a cross-sectional study on water intake behaviors among primary and secondary school students in rural central–western China found that students’ water intake was generally low [36]. These findings highlight the urgency of addressing excessive sodium intake and inadequate water intake, as they can directly impact children’s cognitive function, mental state, intellectual development, and behavior [37,38,39]. Therefore, it is essential to enhance children’s health awareness and promote a low-sodium diet and adequate water intake as healthy lifestyle choices.

Furthermore, this study analyzed the contributions of water intake from fluid and water intake from food to total water intake (TWI) and found that differences in TWI were primarily driven by variations in water intake from fluid. Among the participants, the contribution of water intake from fluid to TWI increased by 23.4% between the first and tenth deciles. This finding is consistent with a 2016 study on water intake in French and British populations, which showed that 80–90% of the variance in TWI was explained by differences in water intake from fluid, with low water consumers not compensating by increasing their intake of water-rich foods [40]. This suggests that increasing water intake from fluid is a key measure to improve total water intake. However, research on the contributions of water intake from fluid and water intake from food to TWI remains limited, and more data are needed to encourage increased water consumption and improved hydration status.

This study also found that boys had a higher dietary sodium intake than girls and were more likely to obtain water intake from food, with a relatively lower proportion of water intake from fluid. Although girls also had a slightly higher proportion of water intake from food compared to water intake from fluid, their water intake from fluid proportion was higher than that of boys. These findings are consistent with multiple existing studies, indicating significant gender differences in dietary sodium and water intake. A study analyzing dietary sodium intake among Chinese adult residents from 2000 to 2015 found that men (6657.5 mg/day) had a significantly higher sodium intake than women (5383.8 mg/day) [41]. A comprehensive survey on residents’ sodium intake in Australia found that men’s average salt intake was 9.6 g/day, and women’s was 6.5 g/day. Additionally, 90% of men and 70% of women exceeded the World Health Organization’s recommended sodium intake (*p* < 0.001) [42]. A community survey on knowledge, attitudes, and practices (KAP) regarding water hygiene found that women had slightly higher rates of regular water intake and consumption of safe water [43]. Currently, no definitive conclusions have been drawn regarding the impact of gender differences on the sensation of thirst. Thus, there may be differences between men and women in terms of voluntary drinking behavior. Further research is needed to identify the causes and develop appropriate measures for improving hydration status. The Chinese Dietary Guidelines (2022) [1] recommend that approximately half of total water intake should come from food and dietary soups. However, the current study results show that both boys and girls had a slightly lower proportion of water intake from fluid compared to water intake from food, falling short of the recommended 50%. This indicates that children’s awareness of keeping sufficient water intake from fluid still needs to be enhanced.

Regarding the relationship between dietary sodium intake and water intake, this study found that as dietary sodium intake increased, water intake from fluid and food increased, and the proportion of children meeting the adequate total water intake also increased. However, the proportion of water intake from fluid decreased relative to total water intake. This finding is consistent with the DASH-Sodium Trial, where the high sodium group had more thirst (*p* < 0.001) and urine output (*p* = 0.007), suggesting higher water intake from fluid [44]. But there’s a lack of such studies in children, and future research could fill this gap. Excessive dietary sodium intake can lead to an electrolyte imbalance and increased extracellular osmotic pressure, stimulating the thirst center and prompting increased water intake [45]. Although increased sodium intake stimulates the thirst center, this response may not always be fully satisfied by water intake from fluid alone. Some studies suggest that when dietary sodium intake increases, the body may regulate water balance through other mechanisms, such as reducing urine output [46]. Additionally, individuals may choose to consume water-rich foods rather than relying solely on water intake from fluid to meet their hydration needs. Therefore, increased an dietary sodium intake does not necessarily lead to a significant increase in the proportion of water intake from fluid and may even reduce it to some extent.

Building on these findings, this study explored the relationship between hydration status and dietary sodium and water intake. The results showed that urine osmolality was associated with water intake from fluid but not with dietary sodium intake. Despite the increased thirst stimulation from higher sodium intake, urine osmolality is not directly related to sodium intake. This is because the body regulates total water intake (including water intake from fluid and food) to maintain an appropriate hydration status [47], thereby keeping urine osmolality within the normal range. When water intake from fluid is sufficient, urine osmolality decreases, indicating adequate hydration. Conversely, when water intake from fluid is insufficient, urine osmolality increases, signaling dehydration and the need for increased water intake to maintain an appropriate hydration status. However, epidemiological and experimental studies have linked low water intake from fluid and markers of dehydration (e.g., increased plasma concentrations of vasopressin and sodium, as well as elevated urine osmolality) to an increased risk of chronic diseases, accelerated aging, and premature death [48]. This suggests that the chronic activation of adaptive responses may be detrimental to long-term health outcomes.

Regarding the strengths of this study, it is the first to investigate the differences in water intake among Chinese children with varying levels of dietary sodium intake and to explore the relationship between hydration status and sodium and water intake. The study used a 7-Day 24 h Fluid Intake Survey Questionnaire and collected urine samples for three consecutive days to account for weekday and weekend variations. Additionally, the use of a weighed dietary survey to assess sodium intake, which does not rely on participants’ memory, provides higher precision compared to other methods such as 24 h recall. Despite these strengths, the study has some limitations. The 7-day duration for drinking water data collection and the 3-day duration for urine and food sample collection may not fully reflect long-term dietary and drinking habits. Additionally, while urine osmolality can promptly mirror short-term hydration status, it may exhibit a lag under normal daily living conditions. Currently, no single gold standard is universally recognized in the literature [4]. Plasma osmolality or Copeptin may represent a better reflection of a single point in time measure. Future research could explore the potential of these biomarkers in evaluating children’s hydration status, and analyze hydration status by integrating multi-indicator results.

## 5. Conclusions

Excessive dietary sodium intake, insufficient water intake from fluid, and inadequate total water intake were common among children. Children with a higher dietary sodium intake tended to consume more water from both fluid and food. Urine osmolality was closely associated with daily water intake from fluid, but not with sodium intake. Interventions to increase children’s water intake from fluid are necessary to maintain an appropriate hydration status and promote health.

## Figures and Tables

**Table 1:** The characteristics of participants.

Characteristic	Total (*n* = 155)	Male (*n* = 87)	Female (*n* = 68)
Age (year), median (IQR)	11 (11, 12)	11 (11, 12)	11 (11, 12)
Height (cm), X ± SD	144.0 ± 7.20	141.7 ± 7.10	146.82 ± 6.31
Weight (kg), X ± SD	36.5 ± 6.99	35.2 ± 7.34	38.2 ± 6.16
BMI (kg/m^2^), X ± SD	17.5 ± 2.26	17.4 ± 2.45	17.6 ± 1.99
Underweight	14.3 ± 1.13 (*n* = 10)	14.6 ± 0.62 (*n* = 9)	11.6 ± NA * (*n* = 1)
Normal	17.2 ± 1.53 (*n* = 129)	16.9 ± 1.17 (*n* = 65)	17.6 ± 1.75 (*n* = 64)
Overweight	21.6 ± 2.51 (*n* = 16)	22.0 ± 2.26 (*n* = 13)	19.8 ± 3.27 (*n* = 3)

Note: Values are shown as the median (M) and quartile ranges (Q), or mean (X) ± standard deviation (SD). * The sample size of this cell is less than 2.

**Table 2:** Sodium intake, total water intake, and water intake from fluid and food with different BMIs and genders.

Characteristic	N	Sodium Intake X (95%CI)	TWI Median (IQR)	Water Intake from Fluid Median (IQR)		Water Intake from Food X (95%CI)	
		(mg/Day)	(mL/Day)	(mL/Day)	%TWI	(mL/Day)	%TWI
Total	155	1647 (1565, 1730)	2039 (1781, 2570)	956 (671, 1219)	45%	1175 (1128, 1222)	55%
BMI							
Underweight	10	1618 (1417, 1819)	2053 (1906, 2862)	991 (641, 1655)	45%	1199 (1075, 1324)	55%
Normal	129	1646 (1556, 1736)	2039 (1768, 2536)	944 (655, 1197)	45%	1171 (1119, 1223)	55%
Overweight	16	1677 (1323, 2030)	2117 (1766, 2866)	1061 (715, 1464)	47%	1193 (1016, 1371)	53%
*p*-value		0.959	0.722	0.660	0.834	0.927	0.834
Gender							
Male	87	1262 (1204, 1321)	2076 (1823, 2579)	893 (654, 1157)	42%	1776 (1664, 1888)	58%
Female	68	1064 (995, 1133)	1985 (1716, 2539)	1004 (708, 1376)	49%	1483 (1370, 1596)	51%
*p*-value		<0.001	0.168	0.185	0.001	<0.001	0.001

Note: data are shown as the mean (X) and 95% confidence interval (95%CI) or median (25th–75th percentile).

**Table 3:** Subjects in different sodium intake groups.

Index	LS_1_ (*n* = 38)	LS_2_ (*n* = 39)	HS_1_ (*n* = 40)	HS_2_ (*n* = 38)	Total (*n* = 155)	*p*-Value
BMI, X (95%CI)	17.62 (16.95, 18.29)	17.13 (16.42,17.85)	17.44 (16.67,18.22)	17.84 (17.07,18.62)	17.51 (17.15, 17.86)	0.570
Gender	12	20	28	27	87	0.001
Male, *n* (%) Female, *n* (%)	12 (31.58%) ^3,4^ 26 (68.42%) ^3,4^	20 (51.28%) 19 (48.72%)	28 (70.00%) ^1^ 12 (30.00%) ^1^	27 (71.05%) ^1^ 11 (28.95%) ^1^	87 (56.13%) 68 (43.87%)	
Water intake from fluid, Median (IQR)	964 (718, 1159)	759 (543, 1050) ^3,4^	982 (720, 1661) ^2^	997 (675, 1201)^2^	956 (671, 1219)	0.031
Water Intake from Food, X (95%CI)	851 (797, 906) ^2,3,4^	1075 (1031, 1119) ^1,3,4^	1224 (1196, 1252) ^1,2,4^	1550 (1484, 1617) ^1,2,3^	1175 (1128, 1222)	<0.001
TWI, Median (IQR)	1827 (1559, 1926) ^3,4^	1796 (1668, 2145) ^3,4^	2228 (1969, 2904) ^1,2^	2550 (2203, 2923) ^1,2^	2039 (1781, 2570)	<0.001
Water intake from fluids/TWI, X (95%CI)	0.52 (0.48, 0.56) ^2,4^	0.42 (0.38, 0.46) ^1^	0.47 (0.43, 0.50) ^4^	0.39 (0.36, 0.42) ^1,3^	0.45 (0.43, 0.47)	<0.001
Meet the adequate daily water intake from fluid, *n* (%)	15 (39.5%)	11 (28.2%)	16 (40.0%)	15 (39.5%)	57 (36.8%)	0.648
Meet the adequate daily TWI, *n* (%)	14 (36.8%) ^3,4^	14 (35.9%) ^3,4^	28 (70.0%) ^1,2^	34 (89.5%) ^1,2^	90 (58.1%)	<0.001

Note: TWI = total water intake. Data are shown as the mean (X) and 95% confidence interval (95%CI) or median (25th–75th percentile). (1, 2, 3, 4): For groups with *p* < 0.05, pairwise comparisons were conducted between the LS1, LS2, LS3, and LS4 groups to further clarify the specific differences between each pair of groups. Groups LS1, LS2, LS3, and LS4 are represented by 1, 2, 3, and 4, respectively. Superscript numerals indicate statistical differences compared with the corresponding groups (*p* < 0.05).

## Data Availability

The original contributions presented in this study are included in the article/supplementary material. Further inquiries can be directed to the corresponding author(s).

## Supplementary Materials

The following supporting information can be downloaded at: https://www.mdpi.com/article/10.3390/nu17071099/s1, Table S1. Average temperature and humidity of the study days; Table S2. BMI limit values of children of different genders and ages.

## Abbreviations

TWI	Total water intake
