# Supplementary material for: Association Between Increased Dietary Sodium Intake and Higher Water Intake from Fluid and Food in Children
Source: Nutrients. 2025 Mar 21;17(7):1099. doi: 10.3390/nu17071099 (PMC11990302; doi:10.3390/nu17071099)
Supplement: Supplementary file 1 [file nutrients-17-01099-s001.zip › nutrients-3519672-supplementary.pdf]

Supplementary Materials:

Table S1. Average temperature and humidity of study days.

	Indoors			Outdoors		
	Dining hall	Dormitory	Classroom	Dining hall	Dormitory	Classroom
Temperature (°C)						
8:00	17.76	16.38	17.04	17.63	16.03	16.49
14:00	21.80	21.72	21.87	22.14	21.58	21.76
20:00	21.09	20.20	21.16	21.08	19.97	20.78
Humidity (%RH)						
8:00	75.82	79.36	78.10	71.73	79.10	77.77
14:00	60.76	60.81	59.68	57.61	59.40	60.10
20:00	72.36	75.50	70.29	71.60	73.13	70.85

Table S2. BMI limit values of children of different genders and ages.

Gender	Age	Underweight	Normal	Overweight
Male	10	≤14.50	(14.50,19.40)	≥19.40
	11	≤15.00	(15.00,20.10)	≥20.10
	12	≤15.50	(15.50,20.85)	≥20.85
	13	≤16.00	(16.00,21.65)	≥21.65
Female	10	≤14.05	(14.05,19.75)	≥19.75
	11	≤14.40	(14.40,20.80)	≥20.80
	12	≤14.80	(14.80,21.70)	≥21.70
	13	≤15.45	(14.45,22.40)	≥22.40
